# Validation of blood culture gram staining for the detection of *Staphylococcus aureus* by the ‘oozing sign’ surrounding clustered gram-positive cocci: a prospective observational study

**DOI:** 10.1186/s12879-018-3412-2

**Published:** 2018-09-29

**Authors:** Yoshiro Hadano, Miwako Isoda, Kazushige Ishibashi, Tatsuyuki Kakuma

**Affiliations:** 1 Department of Infectious Diseases, St. Mary’s Hospital, Kurume, Japan; 2 Biostatistics Center, Kurume University School of Medicine, Kurume, Japan; 3 Department of Clinical Laboratory, St. Mary’s Hospital, Kurume, Japan

**Keywords:** *Staphylococcus aureus*, Gram staining, ‘Oozing’, BacT/ALERT blood culture bottles

## Abstract

**Background:**

*Staphylococcus aureus* bacteraemia is a common and significant infection, associated with high rates of mortality. Therefore, early identification is important for the initiation of appropriate treatment. The objective of this study was to evaluate the accuracy of blood culture Gram staining along with the finding of an ‘oozing sign’ to diagnose either *Staphylococcus aureus* or coagulase-negative staphylococci.

**Methods:**

This single-centre, prospective observational study was performed from May 2017 to November 2017. We used routine blood culture bottles (BacT/ALERT FA and BacT/ALERT SN; bioMérieux, Inc., Durham, NC). Bacterial species were identified and the minimum inhibitory concentration was determined by using the MicroScan WalkAway 96 SI system (Beckman Coulter, Tokyo, Japan). Bottles showing growth were removed, and Gram staining was performed.

**Results:**

A total of 118 samples, including 55 aerobic and 63 anaerobic bottle samples, were analysed. The overall sensitivity of Gram staining was 78.7% (95% CI: 65.8–94.3%), and the specificity was 95.0% (95% CI: 84.7–98.4%).

**Conclusion:**

The ‘oozing sign’ observed in Gram staining may be useful for the rapid prediction of *S. aureus* in BacT/ALERT blood culture bottles.

**Electronic supplementary material:**

The online version of this article (10.1186/s12879-018-3412-2) contains supplementary material, which is available to authorized users.

## Background

*Staphylococcus aureus* bacteraemia (SAB) is a common, significant infection. The 30-day all-cause mortality for SAB is 20–30%, and this has not changed since the 1990s [1]. Early identification is important for quickly initiating an appropriate treatment to prevent persistent bacteraemia, which is associated with a worse outcome [2]. Recently, a new, rapid and reliable identification system, matrix-assisted laser desorption/ionization time-of-flight mass spectrometry (MALDI-TOS MS), was introduced for the diagnosis of microbial pathogens [3]. However, most community hospitals do not have the necessary equipment to perform MALDI-TOS MS due to the high cost of these systems. *S. aureus* is most commonly identified in culture by using the coagulase test, and confirmation requires an additional 24 h [4].

**Fig. 1:**
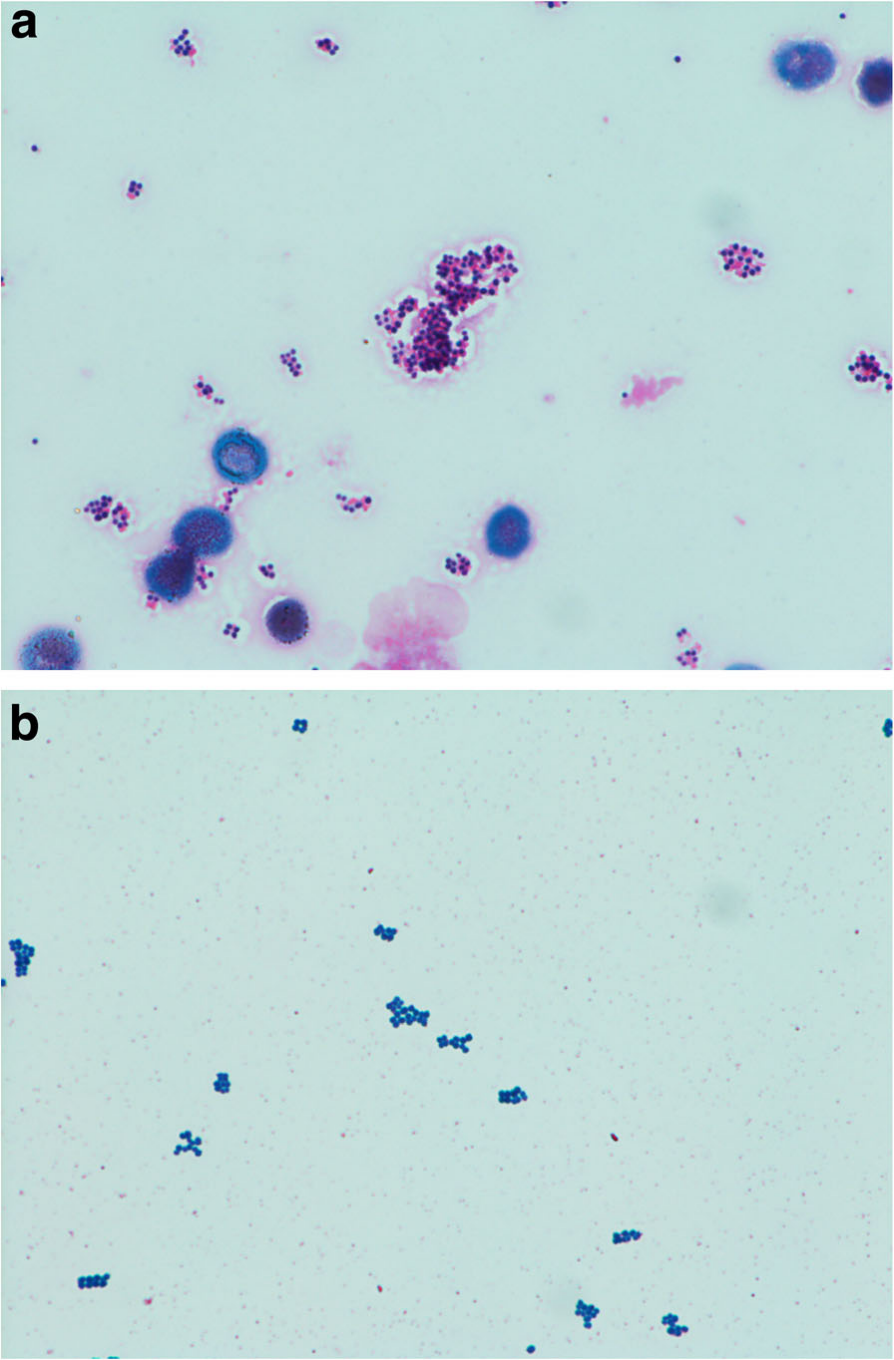
Gram-stained smears from positive BacT/ALERT blood culture bottles showing *Staphylococcus aureus* and coagulase-negative staphylococci. (**a**) ‘Oozing sign’-positive *S. aureus* (Gram-positive cocci in clusters with pink coloured ‘oozing), aerobic bottles. (**b**) ‘Oozing sign’-negative *Staphylococcus epidermidis*, aerobic bottles

Gram staining is a classical method that is convenient and provides considerable information in a short period of time. In our daily experience, a finding of a pink-coloured ‘oozing’ component, which we have termed an ‘oozing sign’, surrounding the clustered gram-positive cocci is sometimes observed for *S. aureus* but for other staphylococci (Fig. 1). Thus, we hypothesized that this ‘oozing sign’ may be a key diagnostic finding that can be used to distinguish *S. aureus* from other staphylococci. The objective of this study was to evaluate the accuracy of Gram staining with the presence of the ‘oozing sign’ to predict the identity of either *S. aureus* or other coagulase-negative staphylococci.

## Materials and methods

This single-centre, prospective observational study was performed at St. Mary’s Hospital (a 1,097-bed acute tertiary care teaching hospital in Fukuoka, Japan) from May 2017 to November 2017. We used routine blood culture bottles (BacT/ALERT FA and BacT/ALERT SN, bioMérieux, Inc., Tokyo, Japan). Bacterial species were identified and the minimum inhibitory concentrations were determined by using the MicroScan WalkAway 96 SI system (Beckman Coulter, Tokyo, Japan). Positive bottles were removed, and Gram staining was performed using Favor method (Nishioka’s method) as described below [5]. Heat-fixed smears on slides were flooded with 0.2% Victoria blue for 1 min and then washed with tap water. Next, the smears were decolourized with 2% picric acid-ethanol, counterstained with 0.004% fuchsin for 1 min, and finally washed with tap water. When the signal became positive between 5 pm and 9 am, Gram staining was performed the next morning. The inclusion criteria were: (a) Gram-positive cocci in clusters and (b) an identification of *Staphylococcus*. The exclusion criteria were: (a) the identification of more than two species and (b) an identification of a species other than *Staphylococcus*. The examiners subjectively assessed Gram-stained samples for the presence or absence of pink oozing surrounding the clustered Gram-positive cocci, the so-called ‘oozing sign’ (Fig. 1). The Gram staining was evaluated by two examiners who knew whether the Gram staining was from either aerobic or anaerobic blood cultures, but were blinded to the identification findings. We then analysed the diagnostic performance of Gram staining of the blood cultures. This study was approved by the St. Mary’s Hospital Research Ethics Committee (Approval No. 17–0101). Blood culture samples were collected daily as part of standard patient care. Since this was an observational study, informed consent was waived.

### Statistical analysis

Precision of the ‘oozing sign’ was evaluated by determining its sensitivity and specificity in comparison to culture-based results. Two test results from aerobic and anaerobic bottles were obtained from each patient, and the two ‘oozing sign’ test results were correlated. To assess the correlation, the generalized mixed model was employed (Additional file 1). Sensitivity and specificity can be modelled with the log link function [6]. Data analyses were carried out in two steps. First, the effect of the covariate was tested, namely the effect of the bottle. The second step was to estimate the sensitivity and specificity with/without covariates. *P* values less than 0.05 were considered statistically significant. All statistical analyses were performed with SAS (version 9.4; SAS Institute, Cary, NC).

## Results

A total of 136 samples were screened for study inclusion. Based on the exclusion criteria, 13 samples with more than two species and five samples with species other than *Staphylococcus*, including *Micrococcus sp.* (*n* = 2), *Enterococcus faecalis* (*n* = 1), α*-Streptococcus sp.* (n = 1), and *Streptococcus parasanguis* (n = 1), were excluded. Finally, 118 bottle samples, including 55 aerobic and 63 anaerobic samples, were analysed. The results of the cultures were as follows: 50 *S. aureus* (26 aerobic and 24 anaerobic samples) and 68 coagulase-negative staphylococci (39 aerobic and 29 anaerobic samples). There was 100% correlation between the aerobic and anaerobic bottles. The overall sensitivity of Gram staining was 78.7% (95% CI: 65.8–94.3%), and the specificity was 95.0% (95% CI: 84.7–98.4%).

## Discussion

To the best of our knowledge, this is the first study on the diagnostic performance of blood culture Gram staining for the detection of *S. aureus* focused on the ‘oozing sign’ using the BacT/ALERT blood culture system. In this study, this ‘oozing sign’ had high overall diagnostic performance, regardless of aerobic or anaerobic culture. This ‘oozing sign’ is a simple and easy method that discriminates between *S. aureus* and other bacterial strains. A previous study showed that criteria based on direct Gram staining characteristics from positive blood cultures were useful for distinguishing *S. aureus* from other staphylococci [7]. This previous study, which was focused on the findings of cell size and cluster characteristics, was able to distinguish between *S. aureus* and coagulase-negative staphylococci. The overall sensitivity was 89%, and the specificity was 98% from BacT/ALERT blood culture bottles, which is the same type of bottles used in our study. For example, they found that *S. aureus* grew as small (< 1 μm irregular clusters containing many bacteria in the anaerobic bottles and as large (≥ 1 μm) clusters in the aerobic bottles. In contrast, our study just evaluated one simple finding, ‘oozing,’ regardless of whether aerobic or anaerobic bottles were used. Our method does not require measurement of cell size, thus, it seems to be more simple, which we believe is advantageous. This method could be used in hospitals with limited resources and outside of Japan.

There are some limitations to this study. Firstly, the results of this study were based on a subjective assessment. In fact, approximately 10% of the assessments were not in agreement. In the future, examiners may need to look at the slides together to obtain the best agreement. Secondly, the findings of this study are applicable only when using the same blood culture bottles and system, as other blood culture bottles may not yield the same result. Thirdly, Gram staining of the positive bottles was not performed at night, and there is a possibility that the ‘oozing sign’ is a time-dependent finding. However, we did not take a possible relationship with time into account. Finally, we do not know what the ‘oozing sign’ is; therefore, further research is needed to characterize this ‘oozing sign’.

## Conclusion

In conclusion, the ‘oozing sign’ observed in Gram staining is useful for rapidly identifying *S. aureus* in BacT/ALERT blood culture bottles. The sensitivity and specificity of this simple finding are relatively high. Therefore, laboratories using the BacT/ALERT blood culture system should learn this simple and easy discrimination method for the rapid identification of *S. aureus* bacteraemia.

## Additional file

Additional file 1: Modelling sensitivity and specificity by the generalized mixed model. (DOCX 12 kb)

## References

[1] van Hal SJ, Jensen SO, Vaska VL, Espedido BA, Paterson DL, Gosbell IB. Predictors of mortality in *Staphylococcus aureus* bacteremia. Clin Microbiol Rev 2012;25:362–386. 10.1128/CMR.05022-11 PMC3346297 22491776

[2] Khatib R, Johnson LB, Fakih MG, Riederer K, Khosrovaneh A (2006). Shamse, et al. persistence in *Staphylococcus aureus* bacteremia: incidence, characteristics of patients and outcome. Scand J Infect Dis.

[3] Idelevich EA, Schüle I, Grünastel B, Wüllenweber J, Peters G, Becker K (2014). Rapid identification of microorganisms from positive blood cultures by MALDI-TOF mass spectrometry subsequent to very short-term incubation on solid medium. Clin Microbiol Infect.

[4] Lowy Franklin D. (2003). Antimicrobial resistance: the example of Staphylococcus aureus. Journal of Clinical Investigation.

[5] Tanaka N, Nagata N, Ooshige T, Takasaki, M. The usability of a simple Gram stain procedure (Nishioka’s method). J Jpn Soc Intensive Care Med. 1997;4:383 [In Japanese].

[6] Margaret Sullivan Pepe (2003). The statistical evaluation of medical tests for classification and prediction. Oxford University press.

[7] Murdoch DR, Greenlees RL (2004). Rapid identification of *Staphylococcus aureus* from BacT/ALERT blood culture bottles by direct gram stain characteristics. J Clin Pathol.

