# Supplementary material for: Validation of blood culture gram staining for the detection of Staphylococcus aureus by the ‘oozing sign’ surrounding clustered gram-positive cocci: a prospective observational study
Source: BMC Infect Dis. 2018 Sep 29;18:490. doi: 10.1186/s12879-018-3412-2 (PMC6162874; doi:10.1186/s12879-018-3412-2)
Supplement: Supplementary file 1 — Modelling sensitivity and specificity by the generalized mixed model. (DOCX 12 kb) [file 12879_2018_3412_MOESM1_ESM.docx]

**Additional File 1**

Modelling sensitivity and specificity by the generalized mixed model.

Let *Y* represent the binary test results, where *Y* = 1 if the test result is positive and 0 otherwise.

Let *D* represent the blood culture results, where *D* = 1 if *S. aureus* present and 0 otherwise.

The sensitivity and specificity of the test can be modelled with the log link function, such that

Log [*P*(*Y_ij_* = 1| *D_i_*)] = *α* + *θX_ij_* + *βD_i_* + *b_i_*, where subscript *i* represent a subject, *j* is the bottle indicator (*j* = 1,2), and *X_ij_* represents a bottle, which is defined as *X_i_*_1_ = 1, *X_i_*_2_ = 0. Parameter *θ* represents the bottle effect, and *b_i_* is the random intercept for subject *i*.

When *D* = 1, the sensitivity is given by exp(*α* + *θX_ij_* + *β*), and when *D* = 0 (1 – specificity) is given by exp(*α* + *θX_ij_*).
